# Calcium Channel Autoantibodies Predicted Sudden Cardiac Death and All-Cause Mortality in Patients with Ischemic and Nonischemic Chronic Heart Failure

**DOI:** 10.1155/2014/796075

**Published:** 2014-03-10

**Authors:** Haiyun Yu, Juanhui Pei, Xiaoyan Liu, Jingzhou Chen, Xian Li, Yinhui Zhang, Ning Li, Zengwu Wang, Ping Zhang, Kejiang Cao, Jielin Pu

**Affiliations:** ^1^State Key Laboratory of Cardiovascular Disease, Physiology and Pathophysiology Laboratory, Fu wai Cardiovascular Hospital, National Center for Cardiovascular Disease, Chinese Academy of Medical Sciences and Peking Union Medical College, No. 167 Bei-Li-Shi Road, Xi-Cheng District, Beijing 100037, China; ^2^People's Hospital, Peking University, Beijing 100044, China; ^3^First People's Hospital of Jiangsu Province, Nanjing 210029, China

## Abstract

The purpose of this study was to evaluate whether CC-AAbs levels could predict prognosis in CHF patients. A total of 2096 patients with CHF (841 DCM patients and 1255 ICM patients) and 834 control subjects were recruited. CC-AAbs were detected and the relationship between CC-AAbs and patient prognosis was analyzed. During a median follow-up time of 52 months, there were 578 deaths. Of these, sudden cardiac death (SCD) occurred in 102 cases of DCM and 121 cases of ICM. The presence of CC-AAbs in patients was significantly higher than that of controls (both *P* < 0.001). Multivariate analysis revealed that positive CC-AAbs could predict SCD (HR 3.191, 95% CI 1.598–6.369 for DCM; HR 2.805, 95% CI 1.488–5.288 for ICM) and all-cause mortality (HR 1.733, 95% CI 1.042–2.883 for DCM; HR 2.219, 95% CI 1.461–3.371 for ICM) in CHF patients. A significant association between CC-AAbs and non-SCD (NSCD) was found in ICM patients (HR = 1.887, 95% CI 1.081–3.293). Our results demonstrated that the presence of CC-AAbs was higher in CHF patients versus controls and corresponds to a higher incidence of all-cause death and SCD. Positive CC-AAbs may serve as an independent predictor for SCD and all-cause death in these patients.

## 1. Introduction

Chronic heart failure (CHF) develops in the setting of left ventricular systolic and/or diastolic dysfunction and is a serious public health problem worldwide with increasing prevalence [1]. Long-term prognosis of CHF is poor and over 50% of CHF patients die within 5 years after diagnosis [2]. A major cause of mortality is sudden cardiac death (SCD) from ventricular arrhythmias [3, 4]. Thus, prediction and prevention of SCD are crucial to management of these patients.

Recently, evidence has been accumulating suggesting that autoimmunity plays a role in the occurrence and progression of CHF [5–7]. For example, *β*1-adrenergic receptor autoantibodies (functioning as receptor agonists) were detected in the serum of CHF patients [8–10] and removal of these autoantibodies has been shown to improve hemodynamic parameters [11]. In addition, antibodies against Na-K-ATPase exerted arrhythmogenic effects and correlated with SCD in certain DCM patients [12]. Immunization of rabbits with sarcolemmal Na-K-ATPase resulted in myocardial hypertrophy due to left ventricular pressure overload and myocardial fibrosis [13]. Therefore, characterizing these antibodies may be helpful in the understanding of CHF pathogenesis.

The L-type calcium channel plays an important role in cardiac excitation-contraction coupling [14]. Dysfunction of the channel often correlates with ventricular arrhythmias (VAs) [15]. Current studies suggest that autoantibodies are directly linked to SCD in DCM patients and calcium channel dysfunction contributes to pathogenesis [12, 16, 17]. Xiao et al. [18] detected the presence of calcium channel autoantibodies (CC-AAbs) in patients with DCM and found that CC-AAbs could prolong action potential duration (APD) and ultimately lead to VT in animal models. They believe that CC-AAbs could serve as a new biomarker for autoimmunity. Therefore, we set out to evaluate whether CC-AAbs could predict prognosis and SCD in CHF patients.

## 2. Materials and Methods

### 2.1. Patients Enrollment

From July 2005 to March 2010, 2096 CHF patients were recruited from 10 hospitals in mainland China. The inclusion criteria were CHF caused by DCM or ICM with NYHA (New York Heart Association) functional class II–IV despite optimized medical therapy and left ventricular ejection fraction (LVEF) ≤45% in DCM and ≤50% in ICM. DCM was diagnosed according to the guidelines for the study of familial DCM [19]. ICM was defined as ≥70% luminal stenosis of at least one major coronary artery diagnosed by coronary angiography and a history of myocardial infarction at least 3 months before enrollment. All cases were excluded if they had malignant tumors, severe liver and kidney dysfunctions or other uncontrollable diseases, pregnancy, and unwillingness to participate in the study.

### 2.2. Control Subjects

834 cases were selected as controls. Of them, 401 cases came from community-based inhabitants who underwent annual health examination and were free of structural heart disease, and 433 cases were hospitalized patients who underwent radiofrequency ablation for supraventricular tachycardia without structural heart disease. The exclusion criteria were the same for controls and CHF patients.

The investigation conformed to the principles outlined in the Declaration of Helsinki and was approved by the Ethics Committee of Fu Wai Cardiovascular Hospital (Beijing, China). All subjects who participated in the study provided written informed consent and reported themselves as Chinese Han nationality.

### 2.3. Serum Sampling and Peptide Synthesis

Blood sample was obtained from the antecubital vein and separated by centrifugation (3000 rpm, Sigma Centrifuge) for 10 min. Serum samples were stored at −80°C until needed for assay. A peptide corresponding to the sequence (residues 2~16) of *α*1c/Ca_V_1.2 of the human L-type calcium channel (V-N-E-N-T-R-M-Y-I-P-E-E-N-H-Q) was synthesized by a commercial source (CL BIO-SCIENTIFIC CO.LTD). The purity of the peptides was determined by high performance liquid chromatography (HPLC) and direct sequence analysis with an automated amino acid analyzer.

### 2.4. Enzyme-Linked Immunoabsorbent Assay (ELISA)

ELISA was used to quantify the CC-AAbs. Briefly, microtiter plates were coated with 100 *μ*L/well calcium channel peptides (5 *μ*g/mL). After incubation at 37°C for 1 hour, the plates were washed with PBS-T 4 times. Nonspecific binding sites were blocked with fat-free milk solution for 1 h and then washed 4 times. The first antibody was added and incubated for 1 h at 37°C. The plates were then washed 4 times, incubated with horseradish peroxidase-streptavidin (Go a Hu IgG-HRP) solution (1 : 500) for 1 h at 37°C, washed 4 times, and developed for 5 min with substrate solution (3,3′,5,5′-tetramethylbenzidine, TMB) in absence of light. The reaction was terminated by stop solution. The optical density (OD) was read using ELISA plate reader (BIO-RAD model 550 USA) at 490 nm wavelength. Autoantibody positive was defined as a ratio (patient OD-blank OD/control OD-blank OD) ≥2.1. The intramicrotiter plate coefficient of variance (CV) level was evaluated using the variation of the negative control from well to well (*n* = 6), whereas the intermicrotiter plate CV level was obtained from different plates. The CVs of intraplates were less than 5%, and CVs of the interplates were less than 10%.

### 2.5. End Point Assessment

The patients were followed up to the end of March 2013 during regular outpatient clinic or through telephone contact. Median follow-up period was 52 months (0.40~92  months). End points included all-cause death, SCD (ICD appropriate discharge counter as SCD), and NSCD (heart transplantation regarded as NSCD). SCD was defined as unexpected death within 1 hour of onset of acute symptoms or unwitnessed death such as during sleep or unexpected death of someone last seen in stable medical condition <24 h with no evidence of a noncardiac cause [20].

### 2.6. Statistical Analysis

Statistical analyses were performed using SPSS 21.0 software (SPSS Inc, Chicago). Continuous values were expressed as mean ± SD, and categorical variables were shown as numbers (%). Student's *t*-test or Chi-square test was used to compare between groups; *P* < 0.05 was considered significant. Person-months of follow-up period started from the date of enrollment to the end of March 2013. Survival analysis in CHF patients was performed. 265 (12.64%) patients were lost to follow up and excluded in survival analysis. Kaplan-Meier curves using log rank test were performed based on presence or absence of CC-AAbs. By using Cox regression, the hazard ratios for time to all-cause death, SCD, and non-SCD from baseline were evaluated.

## 3. Results

### 3.1. Clinical Characteristics

A total of 1831 CHF patients (732 cases of DCM and 1099 cases of ICM) were successfully followed. As shown in Table 1, age and body mass index (BMI) distribution did not differ between CHF patients and controls (*P* > 0.05). Other possible CHF risk factors such as hypertension, hyperlipidemia, diabetes mellitus, premature ventricular contractions (PVCs), atrial fibrillation (AF), mean heart rate (MHR), LVEF, and left ventricular end-diastolic diameter (LVEDD) were more prevalent in CHF patients than in controls (*P* < 0.05). Hemodynamic parameters tested by echocardiography were similar between patients with DCM and with ICM (*P* > 0.05) with a trend towards higher NYHA classification in DCM versus in ICM patients (NYHA II: 21.45% versus 52.96%; NYHA III: 41.80% versus 30.76%; NYHA IV: 36.75% versus 16.28%, all *P* < 0.05). More DCM patients received diuretics and *β*-blockers, while ICM patients took more angiotensin-converting enzyme inhibitors (ACEIs) and calcium channel blockers (CCBs) for hypertension and prevention of coronary artery spasm.

Table 2 lists relevant clinical characteristics between SCD and NSCD subgroups and shows that risk factors such as hypertension, hyperlipidemia, NYHA classification, LVEF, PVCs, and QTc as well as the CC-AAbs did not differ significantly between the two groups (all *P* > 0.05).

Also, we compared characteristics between DCM and ICM patients who were CC-AAbs positive and negative and found no significant differences in age, gender, medications, hemodynamic parameters, and other cardiovascular risk factors attributable to DCM or ICM status (Table 3, all *P* > 0.05).

### 3.2. The Prognosis of Patients with CHF Correlates with CC-AAbs Expression

By the median of 52 months (0.40~92 months) of follow-up, 578 patients (248 cases of DCM and 330 cases of ICM) had died. Of these, 223 patients (102 cases of DCM and 121 cases of ICM) had SCD, while the rest had NSCD. As shown in Table 4, rates of CC-AAbs in DCM and ICM patients were significantly higher than those in controls (5.87% and 4.64% versus 1.20%, both *P* < 0.001). CC-AAbs rates were also significantly higher in all-cause mortality group (9.27% in DCM and 8.79% in ICM) compared to living patients (both *P* < 0.001). Further analysis indicated no difference in CC-AAbs status between SCD and NSCD groups in DCM (12.75% versus 6.85%, *P* > 0.05) and ICM (9.92% versus 8.13%, *P* > 0.05) patients.

### 3.3. Positive CC-AAbs Indicated Higher Risk of SCD and All-Cause Death in CHF Patients

**Figure 1:**
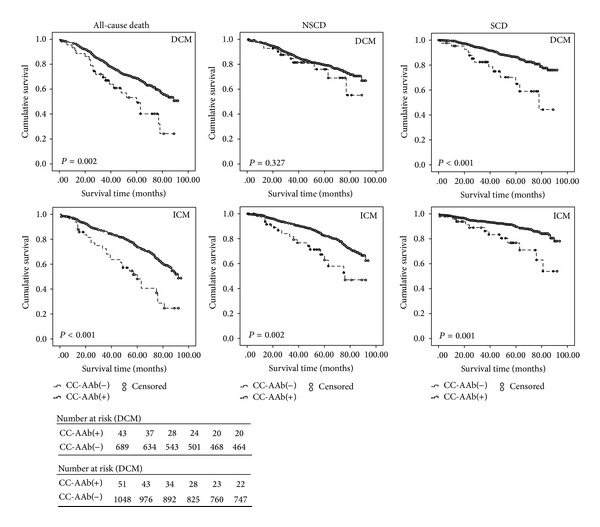
Kaplan-Meier curves for the probability of SCD and all-cause death in patients with DCM (upper panel) and ICM (lower panel) according to the presence or absence of CC-AAbs. Each censored case was marked with a circle dot. Patients with CC-AAbs positive were more susceptible to SCD and all-cause death than patients without carrying CC-AAbs both in DCM and ICM. The CC-AAbs were related with NSCD only in patients with ICM.

Kaplan-Meier curves showed that patients carrying CC-AAbs were susceptible to SCD and all-cause death in DCM (*P* = 0.002 and *P* < 0.001, resp.) and ICM patients (*P* < 0.001 and *P* = 0.001, resp.). However, a significant association between CC-AAbs and NSCD was only found in ICM patients (*P* = 0.002) (Figure 1).

After adjusting for risk factors such as age, gender, BMI, HR, hypertension, diabetes mellitus, hyperlipidemia, NYHA classification, LVED, LVEF, QTc, PVCs, AF, and medications, Cox regression analysis revealed that the presence of CC-AAbs still remained an independent predictor for SCD (HR 3.191, 95% CI 1.598–6.369 for DCM; HR 2.805, 95% CI 1.488–5.288 for ICM) and all-cause mortality (HR 1.733, 95% CI 1.042–2.883 for DCM; HR 2.219, 95% CI 1.461–3.371 for ICM). Consistent with the results of univariate analysis, positive CC-AAbs correlated with NSCD only in ICM patients (HR = 1.887, 95% CI 1.081–3.293) (Table 4).

## 4. Discussion

Our large-scale prospective study demonstrated that more CHF patients had positive CC-AAbs levels than controls, and those positive patients had a high incidence of all-cause mortality as well as SCD. Thus, presence of CC-AAbs could serve as an independent predictor for SCD and mortality. This result is in line with a previous study that showed higher SCD in CC-AAbs positive DCM patients [18].

In recent years, numerous prognostic indicators have been reported to be useful in predicting long-term prognosis of CHF patients: meta-analysis reviews have prompted a few candidates such as brain natriuretic peptide (BNP), ventricular tachycardia (VT), and late gadolinium enhancement (LGE) as predictors of SCD in DCM patients [21–23]. Previous research from us and other groups had also suggested *β*_1_-receptor autoantibodies to be related to SCD in CHF patients [8, 16, 24]. Our present study expanded upon Xiao et al.'s [18] finding that CC-AAbs may be a risk factor for VT and SCD in a small DCM patient group and established that DCM and ICM patients positive for CC-AAbs sustained a 2- to 3-fold risk for SCD.

It is well known that L-type calcium channels play a critical role in signal transduction and electrophysiological activity of the heart [25, 26]. They help to maintain the shape and duration of the action potential plateau phase in ventricular myocytes. When activated by PKA or other factors, more calcium ions flow through the channels, which could trigger activation of the sodium/calcium exchanger and result in intercellular calcium overload, myocyte destruction, and cardiac electric instability [17, 27, 28]. Antibodies against adenine nucleotide translocator (ANT) have been reported to cross-react with a cell surface calcium channel protein to impair cardiac energy metabolism and function through increased intracellular calcium loading [29]. Similarly, autoantibodies against *β*_1_-adrenoceptors have been seen to prolong APD by increasing the L-type calcium currents [16].

Animal experiments have shown that CC-AAbs can produce an agonist effect to calcium-channel function and verapamil, a calcium-channel antagonist, could attenuate immune-mediated myocyte damage [30, 31]. Researchers have suggested a few possible mechanisms by which CC-AAbs could bind to calcium channels: (1) the cell membrane might turn over and result in exposure of intracellular peptide after cardiomyocyte injury happens [32] and (2) autoantibodies could pass through the plasma membrane and penetrate adherent cells through gap junction under certain conditions [33, 34]. Once ventricular myocytes were exposed to affinity-purified CC-AAbs, the APD became prolonged significantly, and this was thought to promote the development of EAD induced ventricular arrhythmias and eventually SCD [18, 35]. These mechanisms may explain why more CC-AAbs were found in CHF patients with SCD.

Our study suggested that CC-AAbs could predict SCD and all-cause death in CHF patients. However, the rate of CC-AAbs observed in our study was lower than that of published data, which reported 48.8% [30]. One possible reason may be our much longer follow-up time, during which the autoantibody activity might have diminished. Nevertheless, we demonstrated CC-AAbs as a positive predictor for SCD and NSCD in ICM patients. Although unreported in the current literature, the prolonged Ca^2+^ current caused by CC-AAbs could delay cardiomyocyte repolarization and increase a risk of developing fatal arrhythmias independent of DCM or ICM etiology of CHF [14]. Multicenter clinical trials have shown that calcium channel antagonist diltiazem had protective effects on the myocardium in DCM patients, particularly during early stage disease [36–38].

## 5. Limitations

It is important to acknowledge some limitations in our current study. First, the ELISA method might have lower sensitivity and specificity compared to other methods such as a complex three-step screening strategy. The effect of CC-AAbs on the prediction of SCD in our study might be underestimated, which would limit its clinical application. Further studies are required to evaluate its diagnostic potential. Second, the CC-AAbs were only tested at baseline and the values immediately before the end point were unknown. Third, a relative high percentage (nearly 13%) of patients was lost during follow-up due to China's recent rapid urbanization. It is important to note that baseline data of these patients were not significantly different. Fourth, we did not follow up the control group, which we should do in the future studies. Finally, our study did not look at the underlying mechanisms behind our findings.

## 6. Conclusions

In conclusion, the presence of CC-AAbs was significantly higher in CHF patients than that in controls. Positive CC-AAbs could serve as an independent predictor for SCD and all-cause death in these patients and may lead to new preventive and therapeutic targets for CHF.

## Figures and Tables

**Table 1:** Clinical data of control subjects and patients with CHF.

Clinical characteristic	Control (834)	CHF			
		DCM (*n* = 732)	*P*	ICM (*n* = 1099)	*P*
Male, *n* (%)	449 (53.84%)	558 (76.23%)	<0.001	885 (80.53%)	<0.001
Age (y)	57.35 ± 12.68	58.86 ± 14.42	=0.052	67.87 ± 10.48	<0.001
BMI	24.63 ± 10.20	24.94 ± 17.05	=0.587	25.02 ± 3.81	=0.453
NYHA class, *n* (%)					
I	834 (100%)	0	—	0	—
II	0	157 (21.45%)	—	582 (52.96%)	—
III	0	306 (41.80%)	—	338 (30.76%)	—
IV	0	269 (36.75%)	—	179 (16.28%)	—
Hypertension, *n* (%)	199 (23.86%)	242 (33.06%)	=0.003	672 (61.15%)	<0.001
Hyperlipidemia, *n* (%)	53 (6.35%)	76 (10.38%)	<0.001	329 (29.94%)	<0.001
Diabetes mellitus, *n* (%)	60 (7.19%)	120 (16.39%)	<0.001	317 (28.84%)	<0.001
ECG and arrhythmias					
MHR (beats/min)	69.95 ± 10.60	79.69 ± 18.89	<0.001	72.57 ± 14.37	<0.001
AF (*n*)	0	177 (24.18%)	—	130 (11.83%)	—
PVC (*n*)	0	192 (26.23%)	—	217 (19.75%)	—
QTc (ms)	412.31 ± 81.21	446.59 ± 102.57	<0.001	444.49 ± 88.67	<0.001
QRS duration (ms)	94.26 ± 57.77	113.64 ± 38.23	<0.001	104.88 ± 42.03	<0.001
Hemodynamic parameters					
LVEF (%)	60.63 ± 9.09	32.91 ± 9.80	<0.001	41.32 ± 8.67	<0.001
LVEDD (mm)	45.34 ± 8.76	66.10 ± 11.96	<0.001	57.59 ± 9.23	<0.001
Medications for CHF					
ACEI, *n* (%)	0	437 (59.69%)	—	710 (64.60%)	—
Diuretic, *n* (%)	0	565 (77.18%)	—	741 (67.42%)	—
Digoxin, *n* (%)	0	483 (65.98%)	—	697 (63.42%)	—
*β*-blocker, *n* (%)	0	570 (77.87%)	—	776 (70.61%)	—
CCBs, *n* (%)	0	24 (3.28%)	—	248 (22.57%)	—
ICD, *n* (%)	0	10 (1.37%)	—	28 (2.55%)	—

Values are mean ± SD or number (%). *P* < 0.05 was considered significant compared with the control group. Premature vascular contraction (PVC) indicated >3000 beats/24 h.

AF: atrial fibrillation; ACEI: angiotensin-converting enzyme inhibitor; BMI: body mass index; CHF: chronic heart failure; NYHA: New York Heart Association; DCM: dilated cardiomyopathy; ICM: ischaemic cardiomyopathy; LVEDD: left ventricular end-diastolic diameter; LVEF: left ventricular ejection fraction; MHR: mean heart rate; ICD: implantable cardioverter defibrillator; SCD: sudden cardiac death.

**Table 2:** Clinical characteristics of CHF patients with SCD and NSCD subgroups.

Characteristics	DCM (*n* = 732)		*P*	ICM (*n* = 1099)		*P*
	NSCD (*n* = 146)	SCD (*n* = 102)		NSCD (*n* = 209)	SCD (*n* = 121)	
Age (years)	57.94 ± 14.67	57.98 ± 14.76	0.982	69.62 ± 10.84	68.78 ± 10.35	0.487
Male gender, *n* (%)	107 (73.29%)	74 (72.55%)	0.959	165 (78.95%)	97 (80.17%)	0.929
MHR (beats/min)	78.66 ± 18.39	79.43 ± 15.74	0.751	75.60 ± 16.39	75.48 ± 16.02	0.942
Hypertension, *n* (%)	48 (32.88%)	31 (30.39%)	0.766	130 (62.20%)	69 (57.02%)	0.643
Hyperlipidemia, *n* (%)	12 (8.22%)	10 (9.80%)	0.693	66 (31.58%)	34 (28.10%)	0.627
Diabetes mellitus, *n* (%)	18 (12.33%)	15 (14.71%)	0.636	76 (36.36%)	29 (23.97%)	0.089
PVC, *n* (%)	39 (26.71%)	30 (29.41%)	0.726	39 (18.66%)	28 (23.14%)	0.430
AF, *n* (%)	47 (32.19%)	21 (20.59%)	0.125	28 (13.39%)	15 (12.40)	0.819
QRS duration (ms)	119.49 ± 40.50	109.79 ± 32.71	0.156	102.38.10 ± 22.29	105.11 ± 24.00	0.921
QTc (ms)	453.81 ± 80.99	447.73 ± 106.04	0.651	443.74 ± 104.56	445.52 ± 62.58	0.862
NYHA						
II	27 (18.49%)	33 (32.35%)	0.052	93 (44.50%)	50 (41.32%)	0.724
III	60 (41.10%)	44 (43.14%)	0.838	69 (33.01%)	48 (39.67%)	0.403
IV	59 (40.41%)	25 (24.51%)	0.064	47 (22.49%)	23 (19.01%)	0.546
LVEF (%)	31.26 ± 9.38	32.62 ± 9.15	0.880	40.09 ± 9.29	38.39 ± 9.33	0.261
LVEDD (mm)	68.15 ± 12.92	65.51 ± 9.85	0.292	58.08 ± 9.45	60.19 ± 9.29	0.151
CC-AAbs	10 (6.85%)	13 (12.75%)	0.153	17 (8.13%)	12 (9.92%)	0.614
BNP (pg/mL)	2127.51 ± 355.79	2123.03 ± 366.57	0.924	2002.27 ± 385.21	1995.56 ± 387.05	0.877
ACEI, *n* (%)	89 (60.96%)	52 (50.98%)	0.410	148 (70.81%)	77 (63.64%)	0.555
Diuretic, *n* (%)	101 (69.18%)	76 (74.51%)	0.709	146 (69.86%)	82 (67.77%)	0.865
Digoxin, *n* (%)	110 (75.34%)	61 (59.80%)	0.260	142 (67.94%)	79 (65.29%)	0.826
*β*-blockers, *n* (%)	108 (73.97%)	78 (76.47%)	0.866	151 (72.25%)	85 (70.25%)	0.874
CCBs, *n* (%)	2 (1.37%)	0	—	34 (16.27)	23 (19.01%)	0.595
ICD, *n* (%)	1 (0.68%)	1 (0.98%)	0.801	4 (1.91%)	2 (1.65%)	0.867

Values are mean ± SD or number (%). *P* < 0.05 was considered significant comparing with NSCD group. PVC indicated frequent premature ventricular beats (more than 3000 beats/24 h). AF: atrial fibrillation; ACEI: angiotensin-converting enzyme inhibitor; BMI: body mass index; CHF: chronic heart failure; NYHA: New York Heart Association; CC-AAbs: calcium channel autoantibodies; DCM: dilated cardiomyopathy; ICM: ischaemic cardiomyopathy; LVEDD: left ventricular end-diastolic diameter; LVEF: left ventricular ejection fraction; MHR: mean heart rate; ICD: implantable cardioverter defibrillator; SCD: sudden cardiac death; NSCD: nonsudden cardiac death.

**Table 3:** Characteristics of CHF patients with CC-AAbs positive and negative.

Characteristics	DCM (*n* = 732)		*P*	ICM (*n* = 1099)		*P*
	CC-AAb(+) (*n* = 43)	CC-AAb(−) (*n* = 689)		CC-AAb(+) (*n* = 51)	CC-AAb (−) (*n* = 1048)	
Age (years)	59.14 ± 13.61	58.84 ± 14.48	0.895	67.69 ± 12.26	67.88 ± 10.39	0.899
Male gender, *n* (%)	34 (79.07%)	524 (76.05%)	0.869	40 (78.43%)	845 (80.63%)	0.898
MHR (beats/min)	79.29 ± 17.98	79.71 ± 18.95	0.883	74.73 ± 18.21	72.46 ± 14.16	0.273
Hypertension, *n* (%)	11 (25.58%)	231 (33.53%)	0.434	30 (58.82%)	642 (61.26%)	0.863
Hyperlipidemia, *n* (%)	3 (6.98%)	73 (10.60%)	0.793	18 (35.29%)	256 (24.43%)	0.191
Diabetes mellitus, *n* (%)	5 (11.63%)	115 (16.69%)	0.452	15 (29.41%)	302 (28.82%)	0.946
PVC, *n* (%)	14 (32.56%)	178 (25.83%)	0.467	7 (13.73%)	210 (20.04%)	0.353
AF, *n* (%)	11 (25.58%)	166 (24.09%)	0.864	4 (7.84%)	126 (12.02%)	0.415
QRS duration (ms)	110.71 ± 35.66	113.82 ± 38.41	0.610	103.25 ± 22.58	104.96 ± 42.76	0.777
QTc (ms)	463.61 ± 67.32	445.50 ± 104.37	0.268	452.41 ± 95.50	444.10 ± 88.34	0.514
NYHA						
II	11 (25.58%)	146 (21.19%)	0.590	25 (49.02%)	557 (53.15%)	0.746
III	18 (41.86%)	288 (41.80%)	0.996	17 (33.33%)	321 (30.63%)	0.768
IV	14 (32.56%)	255 (37.01%)	0.685	9 (17.65%)	170 (16.22%)	0.820
LVEF (%)	33.46 ± 7.63	32.88 ± 9.92	0.718	41.38 ± 8.40	41.31 ± 8.69	0.958
LVEDD (mm)	66.87 ± 8.99	66.06 ± 12.13	0.686	56.10 ± 7.57	57.66 ± 9.31	0.254
BNP (pg/mL)	2139.17 ± 336.68	2089.10 ± 367.98	0.385	1984.44 ± 308.64	1982.79 ± 396.77	0.971
Treatments						
ACEI, *n* (%)	26 (60.47%)	411 (59.65%)	0.958	34 (66.67%)	676 (64.50%)	0.884
Diuretic, *n* (%)	36 (83.72%)	529 (76.77%)	0.710	37 (72.55%)	704 (67.18%)	0.728
Digoxin, *n* (%)	35 (81.40%)	448 (65.02%)	0.340	34 (67.94%)	663 (65.29%)	0.826
*β*-blockers, *n* (%)	37 (86.05%)	533 (77.36%)	0.646	41 (80.39%)	735 (70.13%)	0.817
CCBs, *n* (%)	3 (6.98%)	21 (3.05%)	0.182	11 (21.57%)	237 (22.61%)	0.889
ICD, *n* (%)	2 (4.65%)	8 (1.16%)	0.119	3 (5.88%)	25 (2.39%)	0.147

Values are mean ± SD or number (%). *P* < 0.05 was considered significant comparing with NSCD group. PVC indicated frequent premature ventricular beats (more than 3000 beats/24 h). AF: atrial fibrillation; ACEI: angiotensin-converting enzyme inhibitor; BMI: body mass index; CHF: chronic heart failure; NYHA: New York Heart Association; CC-AAbs: calcium channel autoantibodies; DCM: dilated cardiomyopathy; ICM: ischaemic cardiomyopathy; LVEDD: left ventricular end-diastolic diameter; LVEF: left ventricular ejection fraction; MHR: mean heart rate; ICD: implantable cardioverter defibrillator; SCD: sudden cardiac death; NSCD: nonsudden cardiac death.

**Table 4:** The association between CC-AAbs and the prognosis of CHF patients.

	Control (*n* = 834)		DCM (*n* = 732)			ICM (*n* = 1099)		
	CC-AAb(+)	HR	CC-AAb(+)	HR (95% CI)	*P*	CC-AAb(+)	HR (95% CI)	*P*
Total *n* (%)	10 (1.20%)	1	43 (5.87%)	1	<0.001	51 (4.64%)	1	<0.001
No death *n* (%)	10 (1.20%)	1	20 (4.13%)	1	0.030	22 (3.17%)	1	0.178
All-cause death, *n* (%)	0	1	23 (9.27%)	1.733 (1.042–2.883)	0.034	29 (7.88%)	2.219 (1.461–3.371)	<0.001
Non-SCD *n* (%)	0	1	10 (6.85%)	1.049 (0.483–2.278)	0.903	17 (5.74%)	1.887 (1.081–3.293)	0.025
SCD *n* (%)	0	1	13 (12.75%)	3.191 (1.598–6.369)	0.001	12 (11.57%)	2.805 (1.488–5.288)	0.001

The positive of calcium channel autoantibodies (CC-AAbs) increased hazard ratio. 95% confidence interval (95% CI) after adjustment for age, gender, BMI, MHR, hypertension, hyperlipidemia, diabetes mellitus, QTc, PVC, AF, NYHA class, LVEF, causes of HF, and medications. *P* < 0.05 was considered significant.

## Abbreviations

ACEI: Angiotensin-converting enzyme inhibitors
AF: Atrial fibrillation
BMI: Body mass index
CC-AAbs: Calcium channel autoantibody
CCBs: Calcium channel blockers
CHF: Chronic heart failure
DCM: Dilated cardiomyopathy
ELISA: Enzyme-linked immunoabsorbent assay
HPLC: High performance liquid chromatography
ICM: Ischemic cardiomyopathy
LVEDD: Left ventricular end-diastolic diameter
LVEF: Left ventricular ejection fraction
NSCD: Nonsudden cardiac death
NYHA: New York Heart Association
SCD: Sudden cardiac death
VAs: Ventricular arrhythmias.
